# Deep Learning in the Detection of Disinformation about COVID-19 in Online Space

**DOI:** 10.3390/s22239319

**Published:** 2022-11-30

**Authors:** Kristína Machová, Marián Mach, Michal Porezaný

**Affiliations:** Department of Cybernetics and Artificial Intelligence, Faculty of Electrical Engineering and Informatics, Technical University of Košice, Letná 9, 04200 Košice, Slovakia

**Keywords:** web mining, detection of disinformation, COVID-19, machine learning, deep learning, neural networks, text data processing

## Abstract

This article focuses on the problem of detecting disinformation about COVID-19 in online discussions. As the Internet expands, so does the amount of content on it. In addition to content based on facts, a large amount of content is being manipulated, which negatively affects the whole society. This effect is currently compounded by the ongoing COVID-19 pandemic, which caused people to spend even more time online and to get more invested in this fake content. This work brings a brief overview of how toxic information looks like, how it is spread, and how to potentially prevent its dissemination by early recognition of disinformation using deep learning. We investigated the overall suitability of deep learning in solving problem of detection of disinformation in conversational content. We also provided a comparison of architecture based on convolutional and recurrent principles. We have trained three detection models based on three architectures using CNN (convolutional neural networks), LSTM (long short-term memory), and their combination. We have achieved the best results using LSTM (F1 = 0.8741, Accuracy = 0.8628). But the results of all three architectures were comparable, for example the CNN+LSTM architecture achieved F1 = 0.8672 and Accuracy = 0.852. The paper offers finding that introducing a convolutional component does not bring significant improvement. In comparison with our previous works, we noted that from all forms of antisocial posts, disinformation is the most difficult to recognize, since disinformation has no unique language, such as hate speech, toxic posts etc.

## 1. Introduction

With technological advances and the decreasing costs of communication technologies, more and more people have access to the Internet. Along with this, the demand for information and the amount of data and content on the Internet are growing. However, along with this increased interest, the amount of information intended to manipulate is also increasing. In the past, when information was sold through printed media or disseminated via radio or television, misinformation was less common. However, on various social platforms, content can be created by anyone and easily spread throughout the world at high speed. This type of content is very difficult to control. This gives room for the spread of disinformation and conspiracy narratives. This effect was further accelerated by the COVID-19 pandemic since people started spending more time in the online space. Additionally, the main topic of interest in recent months for most people has been this pandemic, which gave a wide margin for manipulation. The result is many deaths that could have otherwise been prevented.

We focused our research on the detection of disinformation, propaganda, and conspiracy theories about COVID-19. In short, disinformation is distorted or incorrect information that has been deliberately transformed and disseminated in a conscious way. In practice, however, the issue of disinformation is more complex. The concept of disinformation and fake news has become popular in recent years, especially during the 2016 presidential campaign, when the term “Fake News”, popularized by Donald Trump, came to the attention of the public. The very term disinformation is far older, dating back to before the Internet and social networks. Its origin dates to 1923, when it was used by the Russian intelligence service.

*Disinformation* is false or misleading information that is based on some truthful basis. This basis is necessary to make this news more credible. Often, this news is adapted to the cultural context of the target group of users. The authors of disinformation usually have the same goal—to manipulate and influence users. Today, web portals and social networks are mainly used to spread disinformation, which also has the effect of accelerating its spread compared to the past.

*Hoax* is often used as a synonym to disinformation, even though it is more of a subtype of disinformation. Hoaxes have one common feature—they indirectly invite users to share and spread their content. In practice we can often find these messages on social networks with appeals, such as: “share this, before it’s deleted”. With such a plea, the authors are trying to urge the readers to further spread the message, under the premise of containing some secret information, which is being censored by the authorities [1].

*Propaganda* has similar goals as disinformation. It is a form of communication whose goal is to manipulate the public and intentionally shape the thoughts and opinions of the reader. Most often we use this term in connection with authoritarian regimes, but propaganda can also be associated with democratic governments or private entities. In fact, it does not always have to be used with bad intentions. The forms of propaganda sharing are based on foundations which were perfected over many years and have slowly adapted to modern technology. Propaganda tends to be divided into white, black, and grey. Each type is intended to manipulate the public in a different way. White propaganda uses true and objective information. Its goal is to inform and cause a change in public opinion, mobilize, or promote a movement or organization. In modern times, the most well-known form of white propaganda are the many vaccination programmes, in connection to COVID-19. In history, we can also find a form of white propaganda in connection with the selling of war bonds during the second world war, or even army recruitment. The opposite of white propaganda is black propaganda. It uses disinformation, half-truth, and rumors. Black propaganda uses false or twisted information, whose goal is to diminish the relevance and power of their opposition. One of the most interesting examples of black propaganda comes from Great Britain during the second world war. This was the fake German radio station with the name Gustave Siegfried Eins. The voice of this station was Peter Seckelmann, a refugee from Berlin, who under the nickname Der Chef criticized the state of Germany and its government. The last form of propaganda is grey. This form is trying to act neutral, which makes it the least transparent type. It often uses information based in truth, which is however difficult to verify and whose source is unclear. It often spreads through neutral channels [2].

*Conspiracy theories* are today quite commonplace. Literature diverges on their precise definition. Multiple definitions are based on the requirement, that a sort of secret plan exists, in which individuals try to change the natural course of events. For a conspiracy narrative to be successful, the events connected with it must have a society-wide character and be understood as a society-wide threat. That is why the most common target for conspiracy theories are terrorist attacks, wars, epidemics, financial crises, and so on. Even though they are called theories they are often difficult to verify, which makes it incorrect to consider them on the same level as verifiable scientific theories. Common subjects of conspiracy theories are for example the events of September 11, which these theories often attribute to the American government. The generalization of these stories is a conspiracy myth, where as an example we can point to the attempts to put the blame for every negative event on the American government, or even a conspiracy by the Jewish community. In general, these stories and myths originate from a conspiratorial way of thinking, or conspiratorial mentality, which affects individuals or communities with general distrust of influential people or groups [3].

Disinformation is based on different mechanisms, which often exploit errors in judgement, or ingrained attributes that are very natural for humans and are therefore difficult to fight against. Owing to this, the tools of disinformation and propaganda can become effective enough without us noticing them, whether as an individual, or a society. Typical manifestation of disinformation are as follows:

- *Playing with emotions*—stoking of anger, shame, or fear, mainly of the unknown. The response of people to these emotions did not go unnoticed by the companies behind social networks, mainly Meta Platforms owning Facebook, which has lately been criticized for pushing content, which elicits negative emotions. It is clear from their leaked internal analyses, that people are spending more time on the platform if they are served content with a negative subtext. The more time people spend on the platform, the more advertising revenue is generated, which in 2021 reached almost 115 billion dollars [4].
- *Unwillingness to change opinion*—this tendency of human thinking is often misused by the authors of disinformation. They often intentionally present incomplete, edited, or oversimplified information to influence public opinion on a specific event or problem.
- *Confirmation bias*—is an error in judgment, which describes the tendency for people to accept information selectively to support their pre-existing beliefs. This can include an incorrect understanding of connection between two events, or an illusion of correlation [5].
- *Anchoring*—This term has spread mainly in connection with social networks, where false accounts, or troll farms, have been used to anchor users to some opinion. In proactive it manifests itself as a message that gets released into the online space, whose aim is to change the public opinion. The relevancy of this message is then proved using troll farms, whose role is to react positively and vouch for the message in great numbers. One of the most well-known such farm is the IRA (Internet Research Agency) based in Saint Petersburg, Russia, which influences mainly the discourse ground political events. To this day, the most famous documented acts of this agency was their manipulation of American voters during the 2016 presidential elections and their manipulation of United Kingdom citizens in connection with the Brexit referendum [6].
- *Dunning-Kruger effect*—describes the tendency of people to overvalue their own knowledge and experience in areas, where they are the least knowledgeable [5].
- *Filters*—With the advent of social networks, which started to tailor their content for their users, it become easy for online groups to form. Whether this is in the form of Facebook “groups”, or Reddit “Subreddits”. In these groups it is not uncommon for an echo chamber to form [5].
- *Echo Chamber*—People’s opinions and beliefs are strengthened when communicating in a closed group. In such a group, common repetition of information creates positive reinforcement in the absence of dissenting opinions. This can grow into extremism. As an example, we can point to a Facebook group, which came to be during the COVID-19 pandemic in Slovakia named “NEZAOČKOVANÍ” (Unvaccinated). This group started as a place for the exchange of opinions and experiences with vaccination, but over time became radicalized to the point of disseminating threats and encouragement of violence. This group was eventually banned by Facebook due to a breach of the site’s policy [5].

One of the main mechanisms we use to defend ourselves against disinformation is critical thinking. Not everyone, however, has learned critical thinking to the same extent. Many people believe that this means to have a predominantly negative view toward everything, especially inconvenient information. The point of critical thinking is to examine the truthfulness of statements and weighing information, which supports these statements. It is also important to persuade people that it is not bad to trust experts. Recently, people have started to understand academics and experts as a sort of “self-appointed elite ”, which they refuse to trust. Our work wants to be helpful in supporting the truth of the web, so we focused on investigating the possibilities of automatic detection of disinformation. The main contributions of this paper are as follows:

- A new approach to detection of disinformation about COVID-19 based on deep learning.
- Evaluation of the overall suitability of deep learning methods in solving the problem of recognizing disinformation and fake news.
- Comparison of architectures based on the convolutional and recurrent principles.
- Finding that introducing a convolutional component does not bring significant improvement.
- The creation of a dataset containing 27049 texts focused on disinformation about COVID-19. The data set “COVID_dataset.csv” is available at https://kristina.machova.website.tuke.sk/useful/ (accessed on 26 November 2022).

### 1.1. Disinformation about COVID-19

COVID-19 (Coronavirus Disease) is a disease caused by the virus SARS-CoV-2 (Severe Acute Respiratory Syndrome Coronavirus 2). The illness appeared in December 2019, when the Chinese health authorities announced the outbreak of a pneumonia epidemic in the city of Wuhan. Several days later, the genome of a new coronavirus was released to the scientific community, which was at that time named 2019-nCoV. This virus later spread across all continents and caused a global pandemic. Two years later, by the 29th of March 2022 we had a global confirmed total of 481 million cases and more than 6.1 million deaths. The coronavirus pandemic became the everyday topic of conversation for millions of people across the globe, which was followed by a sizeable amount of disinformation, hoaxes, and conspiracy theories. In contract with other topics that give rise to disinformation, in this case the effect was not only about political influence, money, or popularity. The disinformation connected with this pandemic had a sizeable influence on human health and lives. It is therefore a very serious problem.

The victims of COVID-19 disinformation were not only people with tendencies toward conspiracy theories. The disinformation was related to many topics, including testing, vaccination, or even unrelated topics, such as the installing of new technologies during the pandemic (the spread of 5G networks), artificial intelligence, or political events. One of the most common conspiracy theories was the idea, that the pandemic was planned and orchestrated by some group of influential people. According to a survey done in the United States, 71% of the adult population heard about this conspiracy and 20% thought it was likely to be true [7]. 

Other popular disinformation includes a conspiracy theory about microchips being included in testing sets used for diagnosing COVID-19. According to this story, swabs used for these tests included nano-chips, which would enter the body and manipulate human behavior. Later, this conspiracy theory evolved into a form in which the chips were carried by the vaccines, which prevent infection. Another widely spread theory claimed, that the coronavirus was being spread by 5G towers. This resulted in cases of vandalism and violence aimed at destroying the 5G infrastructure. Another common theme for disinformation was suggestions for treating the illness. The recommended treatments included bleach and other chemicals. This also led to overdoses of different medications, often acquired illegally, such as Ivermektine, whose efficacy against COVID-19 was never proved [8].

This form of disinformation led not only to consequences on human health, but also prolonged the pandemic, due to people underestimating the disease, distrusting scientists with their work around vaccines or even intentionally seeking or be infected to gain immunity. These effects worked toward prolonging the pandemic and limiting medical care available for other diagnoses. This indirectly caused deaths and negative health implications for people with other, less urgent diagnoses.

### 1.2. Related Works

There are two different approaches to detecting disinformation. The first is using machine learning methods to train models for the identification of authors of disinformation [9] or to focus on toxicity in texts of conversational content, such as in the article [10] which analyzes hate speech using a web interface with focus on the most popular social networks such as Twitter, YouTube, and Facebook. Then, it is important to decide whether to use strong methods of deep learning or to use ensemble learning, which can work effectively even with weak classifiers. For example, the authors of study [11] have developed Ensemble Learning Scheme using DT (decision trees), SVM, RF (random forest—of decision trees), and KNN (K-nearest neighbors) for sentiment analysis of COVID-19-related comments. We could also focus on the neural networks ensemble models [12], since ensemble models based on classical machine learning methods usually do not achieve the best results [13]. The other direction of research in this field was used in [14] through application of various ensemble strategies to increase the detection performance of a set of models, for example by using random forest voting of fine-tuning for convolutional neural networks.

The study [15] compared machine learning methods with NLP (Natural Language Processing) and offered a short overview of this research area. The results of this study showed that the methods most suitable for the detection of disinformation are convolutional neural networks, recurrent neural networks, GRU, LSTM, and Bi-LSTM. 

Another work used the BERT architecture for the detection of fake news about COVID-19 spread by bots on social networks. In this work the authors used the text of the message, as well as nine additional attributes, such as number of views, friends and the number of hashtags used in tweets. This study achieved results of approximately 85% F1 measure [16]. 

In the work [17] the authors detected fake messages using an architecture based on CNN and LSTM. They used W2V (Word2Vect) for vector representation. Their dataset was composed of the titles and texts of news articles, as well as other articles marked as sources. The goal of the study was to determine whether articles resemble their sources and whether they are related. The authors achieved good results with a relatively simple architecture, namely F1 measure of 97.4%. 

The detection of unreliable users was the topic of the work [18]. In it the authors used a simple model based on CNN and LSTM. Their data were collected from the social network Twitter, namely the profiles of users and their tweets. The dataset contained 4022 profiles. The authors compared their method with SVM and KNN. Using the neural network approach, they achieved 93% accuracy. This was a couple percentage points better than the SVM and approximately 10% better than the KNN.

Moreover, the work of [19] used neural network methods (e.g., BiGRU—bidirectional gated recurrent unit and BiLSTM—bidirectional long short-term memory) and classic methods of machine learning in combination with TF-IDF and GloVe (global vector) representations for cyberbullying detection. They achieved the best results using neural networks—with accuracy and F1 scores as high as –95%.

There are several information technologies that are used for the detection and fight with disinformation. The most common form are educational websites and websites that try to verify information shared on news sites and by public figures (mainly politicians). Since disinformation spreads quite quickly and it is often difficult and time consuming to fact-check it, the scientific community is working on automated models, that can separate false information from truth. These models can then be used to uncover disinformation. In Slovakia, there are several such web portals. For example, the website “demagog.sk” (accessed on 28 October 2022) marks the statements of Slovak politicians by their truthfulness. The statements are classified into categories (true, untrue, misleading, unverifiable). Another portal “konspiratori.sk” (accessed on 28 October 2022) focuses on websites that spread questionable content. These websites are marked with a number from 1 to 10, where 10 is the worst possible rating. The portal “blbec.online” (accessed on 28 October 2022) focuses on information posted on the Slovak section of the social network Facebook. This portal collects data from questionable profiles. The last one we will mention is “zvolsi.info” (accessed on 28 October 2022). It is an educational web, which tries to teach media literacy and how to work with information. The authors of this website also organize talks in schools aimed at teaching these skills. 

There are many similar foreign web portals, which help a user to recognize fake news as TIM (Tools for Innovation Monitoring) “timanalytics.eu” (accessed on 22 November 2022) and Ecanvasser—fake news detector “ecanvaser.com” (accessed on 22 November 2022). The European Data Protection Supervisor provides user with information about fake news and their dissemination and information about data protection “edps.europa.eu” (accessed on 22 November 2022). There are also 13 AI-Powered Toolls for Fighting Fake News on “thetrustedweb.org”, which support the truth of the social web (accessed on 22 November 2022). Another web portal—RAND Corporation obtains tools that fight disinformation “rand.org” (accessed on 22 November 2022). For facts check, there is Google toolbox Fact Check Explorer “toolbox.google.com” (accessed on 22 November 2022). Fact checking also includes the web portal “open.edu” (accessed on 22 November 2022), which contains social media fact-checking strategies. 

## 2. Materials and Methods

When solving the task of detecting disinformation, specifically fake news about COVID-19 in text data, we needed to use both natural language processing methods, as well as machine learning methods.

### 2.1. Data Description

We wanted to use text data in Slovak at first. The Slovak language is a so-called Low Resource Language. It means that there are not many sources of data in Slovak. We found it quite difficult to extract enough of such data. This was the reason why we finely decided to use data in English.

We prepared the dataset by processing and joining together more suitable datasets downloaded from “kaggle.com” (accessed on 28 October 2022). The final dataset consisted of two columns: one containing the text of news articles and the second was annotation—“1” for disinformation and “0” for truthful news. The sources for our dataset were the following:

- COVID-19 Fake News Dataset—contained 33 files in the form of .csv obtained from the newspaper titles and statuses from the social network Twitter. Those files did not contain text, only ID tags, so we had to extract the texts manually. Those data were cleaned from unnecessary information and were labelled. There was also an attribute named “fact_check_url” which means that the data also contains a source. The source is usually a respected organization, such as WHO, medical, or scientific media. The final version of the dataset contained 4504 training examples.
- COVID disinformation dataset contained 4 files with data divided into train, validation, and testing sets, which was an advantage. After pre-processing, this dataset contained 12,005 items.
- Disinformation dataset was like the previous. It was labelled but in the opposite way to what we needed, so the labelling had to be checked and revised. In the end there were 10,326 training examples.
- Real and fake news contained 4870 examples. Labelling was there in the form of a number, but also as a string. Some attributes and unnecessary information were removed.

After joining all four sources of data and removing duplicate items we obtained 27,047 examples, of which 54.7% were disinformative (1) and 45.3% were truthful (0). These data were pre-processed in the following way:

- Tokenization
- Transformation to lower case letters
- Removing stop-words
- Stemming.

After pre-processing other duplicates were discovered. After removing those duplicates, the final data set contained 25,870 examples. 

Then we created a vector representation of the text data using the “Gensim” library of the Python programming language. We have created more W2V (Word2Vect) representations with various values for “*length*”—30, 64, 120, and 200. The higher values for the length of the vector mean higher precision, but also more danger of over learning, which would cause lower precision on the testing set. For all testing vector lengths, we created SG (Skip-gram) and CBOW (Continuous Bag-of-Words) representations. The Skip-grams could generally achieve better results, because they are more sensitive on the less frequent words. 

More hyper parameters needed to be set for the W2V creation. One of them is the length of the context “*window*”. We set the “*window*” to 10 words, because our text examples were rather short—newspaper titles, short posts, etc., and each word may be important. 

The parameter “*min_count*” was set to 2. That means, that the words with a frequency of occurrence of 2 or less were omitted. All models were trained within 15 epochs. We have generated several models of W2V representations and saved them in the form of .txt for use in the creation of Embedding layers in neuron network model training. The best was the W2V representation with the length of 64 words. 

### 2.2. Natural Language Processing

Due to the rapid development of the social web, a great amount of short text data was available. As a result, we needed to process non-structural text data. It led to the development of subfields of artificial intelligence, namely text mining and natural language processing (NLP). One of the tasks of NLP is transforming the text into numerical representation, which is more suitable for text data processing by software. This is the task of vectorization. Sometimes we need to do this transformation without losing the information about the context of the word. This problem is solved by Word2vec [20]. 

**Figure 1:**
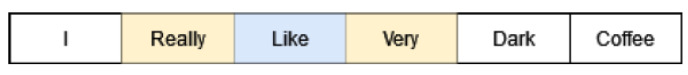
Illustration of the window for creating a context of the word “like” through W2V.

Word2vec (W2V) is one of the most used tools for text vectorization. This model is in principle a shallow neural network based on one input layer, one hidden layer and one output layer. It uses unsupervised learning; whose input is a pre-processed corpus of words. Compared to previous methods for word vectorization, W2V has the advantage that it can set the vector of individual words based on the context words surrounding it. Before training, it is possible to select one of two architectures for determining the vectors for words. The first one is Continuous Bag-of-Words (CBOW). This architecture works on the idea of predicting a word’s vector based on the surrounding words. In the input we look at a certain number of surrounding words, which can be set as “parameter window” and the output is the chosen word. Figure 1 illustrates the context of the word “like” in the sentence “I really like very dark coffee”. The sum of the vectors of these words is averaged and used as input, while the output is the vector of the word we are looking for.

**Figure 2:**
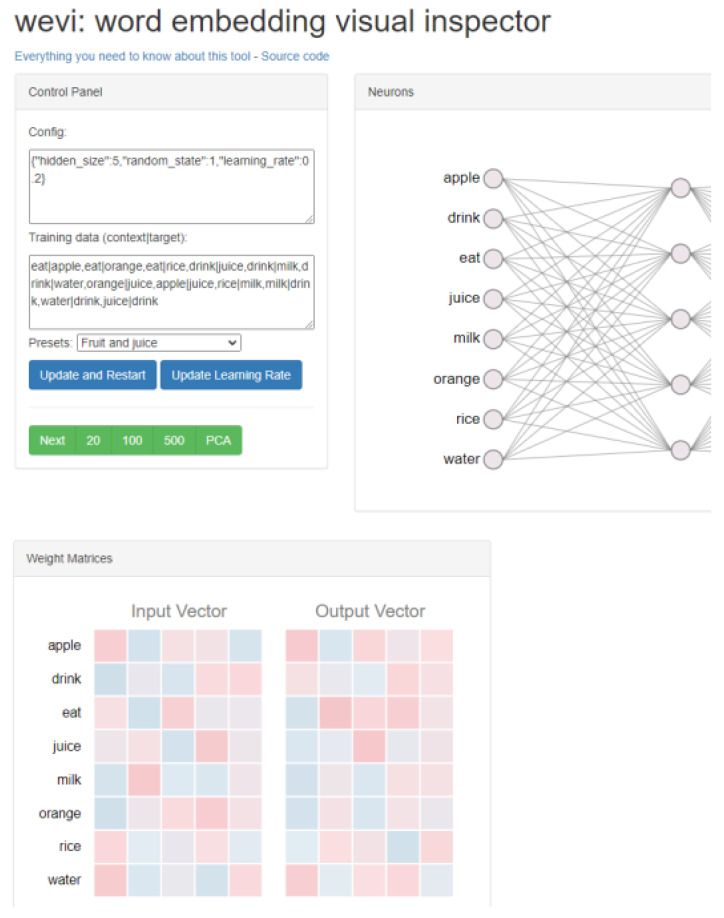
Illustration of the web tool “wevi”.

The second possible architecture is the Continuous Skip-gram. This architecture is different, in that it selects the surrounding words based on the input word [21]. For easier understanding of Word2Vec, there is an interactive tool called wevi (word embedding visual inspector). In this tool it is possible to choose the architecture (CBOW, Skip-gram), the number of neurons in the hidden layer (vector size), insert training data and the number of training epochs. Wewi also displays the changes in weights and the embedding of words into a 2D space through a PCA decomposition. This tool is illustrated in Figure 2.

### 2.3. Used Methods of Deep Learning

Based on our previous works [9,13] we focused on the most successful methods of deep learning, namely convolutional as well as recurrent networks. 

*Convolutional neural network* is a type of deep neural networks, which function by using the mathematical function convolution, which can be understood as multiplying two functions. For this, the network uses convolution filters, also called kernels. These filters are matrices, usually square, which are used in convolutional layers. A CNN also contains pooling layers, which reduce the size of their input. Through a combination of these two types of layers we can reduce the total number of parameters compared to a fully interconnected network, while reducing noise. For some applications, the reduction in resolution in each convolutional step could however be unsuitable. Any neural network that contains at least one convolutional layer can be considered a convolutional network [22]. 

**Figure 3:**
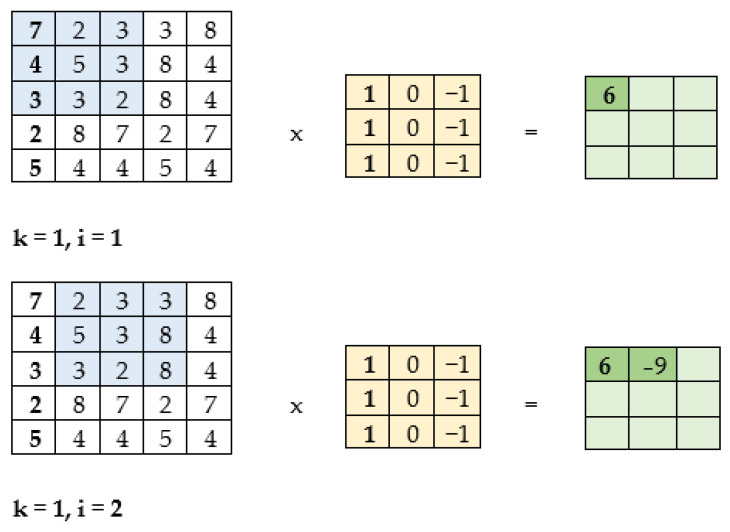
An example of the operation of convolution for image processing with the parameter “*stride*” = 1.

CNNs were initially used for image processing. For this purpose, square convolution filters with odd dimensions were used, which move through the image with a certain stride *k*, most often *k* = 1. This is illustrated in Figure 3.

**Figure 4:**
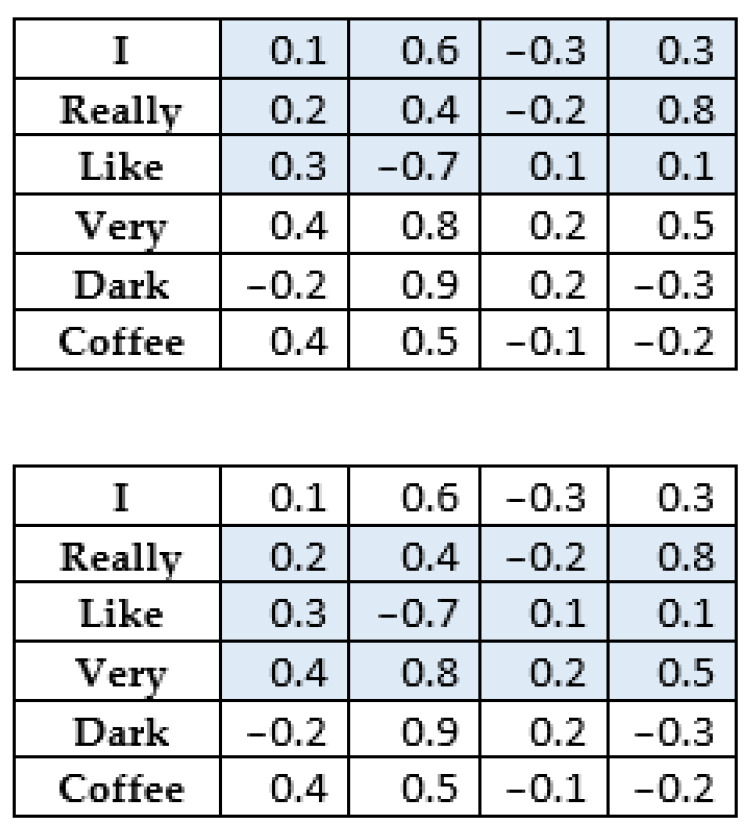
An example of the convolutional operation in text processing. The 1D convolution with the length “*window*” = 3 is illustrated.

The idea was adapted for the task of text data processing. With text we use 1D convolution. This means, that the kernel in this case moves only in one direction, while covering the full length of a word vector in the other dimension. So, if the vector length is 64 and the kernel spans 5 words, then the size of our kernel in 1D convolution is 64 times 5, as illustrated in Figure 4.

The result after the first convolution is one column vector. However, this vector is shorter than the number of words in the input due to the attribute of the convolution—a resolution reduction. This problem is usually solved by the so-called “padding” in the next voting layer—a zero vector is added to the beginning and end of the input string. This type of padding is usually referred to as “*same*”, that is, the input is the same size as the output. There are also other options. There are several convolutional filters in each convolutional layer. These filters, i.e., the values contained in them, represent the parameters of the convolutional layer. The number of these filters is a hyper parameter of the network, which is set for the network by the user. Thus, passing one filter through the input vectors gives us one output vector after convolution. We get one vector for each filter. The result after passing all the filters is a matrix. The next step is the use of a pooling layer, in which we reduce the number of sings, when, similar to convolution over pictures, in a certain window, either the highest value for the vector in in the window (Max Pooling) or the average value for the vector in the given window (Average Pooling) is selected. The window size can also be set as a “*pool size*” hyper parameter. As with the convolutional layer, so also with the pooling layer, a “*stride*” step is set, in which the pooling window moves along the signs—words selected from the convolutional layer [22]. 

**Figure 5:**
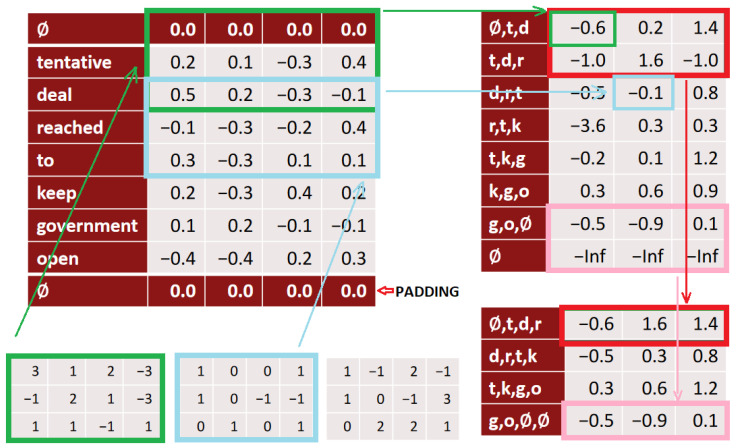
The process of 1D convolution with 3 filters and a pooling layer [23].

The whole process of 1D convolution and subsequent voting in the pooling layer are shown in Figure 5. On the upper left are the input vectors for individual words that form the input matrix for the convolution layer. Individual convolution filters pass through these vectors (bottom left), which give us one output vector for the entire input sequence of vectors (one column of the matrix, top right). Vectors of three words (kernel size is three) are transformed into one number. If we have three filters, as in Figure 5, we get three values, that is, the final matrix contains three vectors. Second step after convolution is pooling (matrix on the bottom right).

In this way, in several layers, the signs are reduced, which we transfer to a fully connected layer, which then enters the output neuron (or more when classifying into several classes), which determines the result of the classification [23].

*Recurrent neural networks (RNN)* can work with the text as a sequence of words, where the order of the words and their context are considered. Thus, the text is not taken as a “bag of words” without relation between them, as is the case with other machine learning methods and with simple neural networks. The most known recurrent network is LSTM (long short-term memory), which are also a specific network among all recurrent networks, because they can re-store information for a longer time and that is why they can process longer sequence of words. 

**Figure 6:**
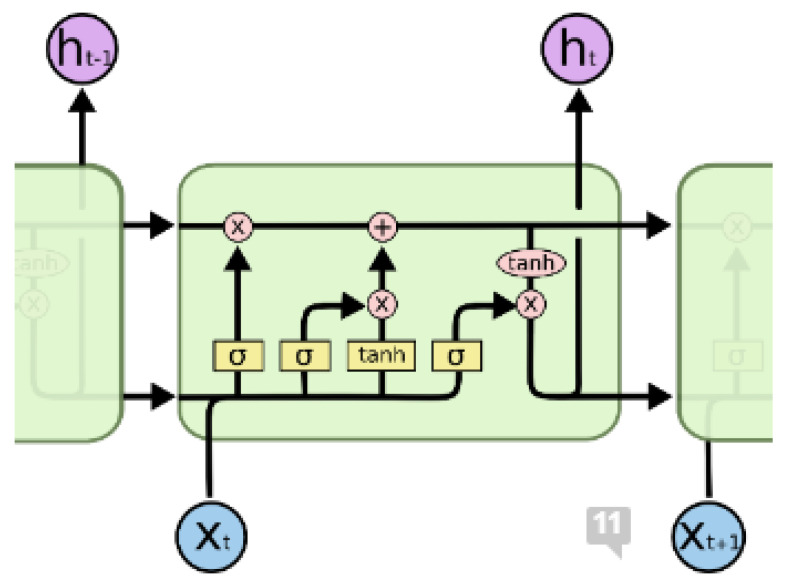
The process of training the LSTM network [25].

LSTM networks are composed of repeating modules (LSTM blocks), in the form of a chain. These blocks have four layers in which individual parameters of the neural network are trained. These layers interact with each other in different ways [24]. Figure 6 illustrates the principal scheme of the LSTM network. The yellow squares are individual layers of the LSTM network, i.e., layers that contain trainable parameters. The pink circles represent the operation of addition or multiplication of vectors (Hadamard multiplication). The black lines represent the flow of vectors.

The basis of LSTM is a horizontal line through which a vector passes between individual blocks. Using this line, information passes through the entire structure of the LSTM network. There are gates in individual cells. These gates are used to remove or add information to the state of the block. Information (vector) passes through these gates, which are composed of neurons with a sigmoidal activation function. Depending on the value of the output on these neurons, so much information passes through it, while 0 means that no information passes through the neuron (i.e., the gate), and 1 means that everything passes through the gate. Each LSTM block contains three such gates (input gate, forget gate and output gate), which are used to adjust the state in which the individual blocks are located. The first gate decides what information is to be forgotten, or what part of it [24].

There are several variations of the LSTM network that use this basis, but with some variations in some parts of the block. One of the most famous is GRU (Gated Recurrent Unit). This variation combines input gate and forget gate into one gate [25]. This means that the GRU is simpler because it has only two gates in total.

## 3. Methodology of Research

Our methodology of building and evaluation of models can be described by following methodology:

- Dataset creation and division
- Creating of an embedding layer
- Training CNN model
- Training LSTM model
- Training CNN+LSTM model
- Models’ evaluation

For neural networks training it is important to divide dataset on training, validation, and testing subset. We selected 64% of data for training, 16% for validation, and 20% for testing. For splitting of data, we used tool “*seed*” from the library “sklearn”. Then the data were split in the same way for each repetition of data division. 

### 3.1. Embedding Layers Creation

It is possible to use a pre-trained model for increasing the efficiency of training. We used one such available model “*Word2vec Google News 300*” containing vectors for 3 million words.

We used our dataset, whose creation and description are in Section 2.1. “Data description”. This dataset was vectorized, and, in this way, the embedding layer was created. Neuron networks need to have in input all sentences of the same length. So, the sentences should be shortened or divided into more sentences. We had short texts in our dataset, so it was not necessary to reduce number of attributes (words). On the contrary we added zero values into vectors of too short texts. For creation of embedding, we have used W2V in two various representations—Skip-gram (SG) and Continuous Bag-of-words (CBOW). 

### 3.2. Creation of CNN Model

First, we tried to learn a CNN model with a simple architecture. We chose three convolutional layers. After the first two, there was a pooling layer with parameters “*pool_size = 2*” and “*padding = same*”. The pooling layer “*GlobalMaxPool*” was located after the third. Behind this layer was a fully connected layer with 128 neurons, and finally an output layer with one neuron and a sigmoidal activation function. The other layers used ReLU as an activation function. We chose “*binary cross-entropy*” as the loss function. We also used the Adam optimizer. Accuracy was used as a metric of effectivity of the model. We trained the model during ten epochs, we chose a batch size of 16, and we chose a learning parameter of size 0.0001 (1 × 10^–4^). After training, we visualized the course of accuracy on the validation and training set, as well as the course of the error function during training. It was clear from the resulting graph that the model is being overlearned considerably and after the second epoch the error on the validation set did not decrease, on the contrary it increased, so there was a divergence in the validation set. The model had a significant tendency to overlearn quickly. 

We therefore proceeded to simplify the complexity of the model and removed one convolutional and voting layer. However, the result was the same even after changing the hyper parameters, whether with the original or simplified architecture. So, we moved on to introducing dropout layers. After the fully connected layer, we introduced a dropout with a probability of 0.5, which is used most often in practice for this layer, and probability 0.2 for voting layers. Subsequently, also by changing the hyper parameters of the model, we got the final topology, which is represented in Table 1. To initialize network layers (convolutional and fully connected), we used Xavier uniform initializer [26] to initialize kernel weight matrices while bias vectors were initialized to zeros. We reduced the number of convolutional filters, reduced the learning parameter to 0.00001 (1 × 10^–5^), and reduced the number of neurons in the fully connected layer. Since we significantly reduced the learning speed of the model, we proceeded to increase the number of epochs to 120. In this way, we gradually got a model that was able to work with all W2V representations, while it did not overlearn. 

We have successfully trained the model on this topology with all W2V vector lengths and with the same hyper parameter settings, except for using the Google News W2V model. This model had a tendency for the error function to diverge on the validation set, so we adjusted the batch size from 30 to 60, when this problem was removed.

For this resulting model, we created a confusion matrix, from which we subsequently calculated other measures—Precision, Recall, F1, and Accuracy. The achieved results of this CNN model on the testing set are presented in Table 2.

Table 2 shows that with CCN we achieved the best results with the model trained on the vector representation of W2V texts using Skip-gram and the size of the vectors 200. This model achieved Accuracy 0.8533 on the testing set. The F1 measure, i.e., the harmonic mean of Precision and Recall, reached a value of 0.8679, or 86.79%. This confirmed our hypothesis that it is better to use a larger size of vectors, however, this may lead to overtraining of the model, which was reflected in a higher accuracy compared to W2V Google News, which has a length of 300. It can also be seen that using the Skip-gram model is better as the use of CBOW, which reached about 1.4% lower value of the F1 metric.

### 3.3. Creation of LSTM Model

We built the LSTM topology similarly to the CNN model topology. The model consisted of an input layer, an embedding layer, an LSTM layer composed of 100 LSTM blocks (we normalized the size of the input sequence to 100 tokens). The LSTM layer was followed by a fully connected layer with 30 neurons. After this layer, we included a dropout layer with 30 neurons. The last layer was only the output layer consisting of one neuron with a sigmoidal activation function. In the first run, we kept the hyper parameters of the network the same as in the CNN case. This means that the batch contained 30 samples, the number of epochs was 120, we left the learning parameter at 0.00001 (1 × 10^–5^). We kept the error function binary cross-entropy, we also used the same Adam optimizer. To initialize network layers (LSTM and fully connected), we used Xavier uniform initializer to initialize kernel weight matrices, Orthogonal initializer [27] to initialize recurrent kernel weight matrix, and zeros to initialize bias vectors. 

The first results showed that the model shows signs of overlearning, but not as much as with CNN. Therefore, we did not change the topology of the network, but only the hyper parameters. We increased the batch size to 48, reduced the number of epochs to 90. After training, it was obvious that the model’s properties improved, but there was still a slight overtraining. Finally, we reached the number of epochs 60, and the batch size of 60. We did not change other parameters. This model was trained as on the first W2V Skip-gram model with a vector size of 64. For all other vector representations, the model behaved the same, so it was not necessary to change the hyper parameters of the model further. The final topology of the LSTM model is shown in Table 3.

We evaluated the LSTM model on the test set in the same way as with CNN, we created a confusion matrix, from which we subsequently calculated other metrics. The results are presented in Table 4. In this case, the model with the size of W2V vectors 200 also turned out to be the best, but not with the Skip-gram type, but with CBOW. The F1 rate was 0.55% higher with CBOW compared to Skip-gram. The CBOW representation had an F1 rate of 0.8741, or 87.41%. At Skip-gram it was 0.8686, in percentage 86.86%. Overall, the difference in F1 rate between the various lengths of individual vector representations was very small. The vectors from the Google News model achieved the lowest accuracy.

### 3.4. Creation of CNN+LSTM Model

The properties of the LSTM and CNN architectures allow us to combine these approaches into a hybrid neural network. Such a connection can led to an improvement of the model by a few percent. We therefore decided to try this approach for our task as well. We chose an architecture in which we included one convolutional layer after the embedding layer, followed by a pooling layer and a dropout layer. We chose the same number of convolutional filters as for CNN, i.e., 30, and their size was 5. We also left the padding “same”. In the pooling layer, we chose the size of the voting window as 2. In the dropout layer, the probability parameter was set to 0.2. We kept the other parts of the architecture the same as in the case of the LSTM network. Similarly, to previous cases, to initialize network layers (convolutional, LSTM and fully connected) we used Xavier uniform initializer to initialize kernel weight matrices, orthogonal initializer to initialize recurrent kernel weight matrix, and zeros to initialize bias vectors The topology of the CNN+LSTM network is described in Table 5. 

Training this architecture was more complex because each vector length needed to adjust the hyper parameters. We set the batch size different for each vector length. For vector length 30 it was a batch of size 30, 48 for vector length 64, then 60 for vector length 120, and 72 for vector length 200, which was the same as in the W2V Google News 300 representation. The hyper parameters settings in this way showed no signs of overlearning, except for a slight overlearning at the largest vector length, i.e., 300 for W2V Google News. We did not manage to optimize the model with this embedding layer very well even after adjusting its other hyper-parameters, it still showed signs of overlearning on the training data, or the error function reached the local minimum where the model converged. This model showed weaker results, and thus its performance in the state when it was only slightly overlearned was the weakest. The best results were again shown by the model with a vector length of 200 and the W2V Skip-gram learning method. This model had an F1 rate higher than Google News W2V by approximately 3.5%, at the level of 86.72%. We evaluated the models in the same way as with the previous architectures. Results of experiments with topology CNN+LSTM presented in Table 5 are in Table 6. 

## 4. Discussion and Conclusions

All trained models achieved relatively similar results. The best model is a model based on the LSTM network, using W2V with a vector length of 200 (F1 = 0.8741). In the case of the CNN architecture, we achieved the best result of F1 = 0.8679, also using a vector representation of length 200. With the hybrid architecture using the combination of CNN+LSTM, we achieved an almost identical result of F1 = 0.8672. The results of the best models from each topology are summarized in Table 7.

The best result from our models from Table 7 is compared to best results in related works in Table 8. We can see that detection of disinformation is a more complex problem than others, for example detection of fake news. 

Since results provided by the trained models are similar, it is useful to carry out significance analysis of the results to determine whether the results are from the same or from different distributions. We used well-known *t*-test [28] with null hypothesis assuming that there is no difference between means (i.e., results are drawn from the same distribution). Results of significance analysis of achieved results (represented by harmonic mean F1) are presented in Table 9.

Basically, two conclusions (recommendations) can be drawn:

- Convolutional- and sequential-based topologies provide different results as seen from LSTM vs. CNN/CNN+LSTM pairings (and sequential principle should be preferred to convolutional);
- Mixing convolutional and sequential principles together into a hybrid architecture does not provide additional classification quality as seen from CNN vs. CNN+LSTM pairing (classification performance of the hybrid topology remains on the same level as for CNN topology).

Computational complexity of a deep model depends on various characteristics such as number of data points, number of learnable parameters of model structure, initialization of parameters, learning hyperparameters, etc. Since we dealt with different network structures, our concern was to find suitable network topologies. Although our search was primarily driven by classification quality, it was related with network complexity as well. We tried to avoid creating overparametrized nets with the number of parameters too large with respect to the number of data points available for training. After we recognized our network structure as overparametrized (when model was identified as overlearned), we subsequently simplified it toward final topologies.

Since reducing complexity was not our primary goal, complexity of our topologies may be decreased further. It could be one possible direction of further research—to consider for example methods for sparsifying trained neural network, structure pruning, meta learning when model learns useful information about the learning process or low-rank compression replacing a large matrix multiplication with two or more smaller matrices (e.g., replacing full convolution by depth-wise separable convolution [29]).

It was also confirmed that models using longer vectors achieve better results; however, only to a certain extent, which if exceeded, the accuracy of the models decreases. To verify the hypothesis of decreasing the accuracy of models using longer vectors (particularly a length of 300 provided by Google News representation), we performed outlier analysis. For each network topology, a list of standardized residuals was calculated in relation with used representation length. Results of this analysis of achieved results (represented by harmonic mean F1) are presented in Table 10.

From the table it is obvious that the largest W2V representation really provides values of standardized residuals quite close or exceeding absolute value 2 (especially in connection with topologies incorporating sequential layers). Since some researchers consider value 2 as threshold for identification of outliers, usage of representation length of 300 can be considered as too underperforming and should not be recommended.

It is also clear that in most cases the use of Skip-gram architecture achieves better results when learning W2V models. According to significance analysis based on *t*-test, SG and CBOW representations provide results significantly different. Therefore, SG representation for W2V models should be preferred.

Interestingly, with the LSTM architecture, there were very small differences in the use of individual vector lengths. If we neglect the Google News 300 representation, the difference between the best and worst model was only about 1.5% for the F1 metric. When using the CNN architecture, this difference was higher, reaching a value of approximately 2.6%. It was similar when combining both architectures. When not considering the Google News 300 representation, the difference was at the level of approximately 2%. For Google News 300 it was about 3.5%.

With further optimization of the hyper parameters, or by expanding the dataset, or using augmentation of the current dataset, we could probably achieve a few tenths of a percent better results. Future research could also focus on using capsule CCNs, or the use of other models for vector representation such as FastText or GloVE.

## Figures and Tables

**Table 1:** Topology of the CNN model.

Layers	Parameters
Input	-
Embeding	-
Conv1D	30, 5, ReLU
MaxPooling	3, 2, same
Dropout	0.2
Conv1D	30, 5, ReLU
MaxPooling	3, 2, same
Dropout	0.2
Conv1D	30, 5, ReLU
GlobalMaxPooling1D	-
Dropout	0.2
Dense	64, ReLU
Dropout	0.5
Output	1, sigmoid

**Table 2:** Effectivity of CNN model with the topology presented in the Table 1.

CNN	Precision	Recall	Accuracy	F1
64 SG	0.8651	0.8651	0.8441	0.8651
64 CBOW	0.8448	0.8448	0.8264	0.8448
30 SG	0.8667	0.8667	0.8423	0.8667
30 SBOW	0.8423	0.8423	0.8156	0.8423
120 SG	0.8622	0.8619	0.8483	0.8620
120 CBOW	0.8437	0.8568	0.8317	0.8502
200 SG	0.8714	0.8645	0.8533	0.8679
200 CBOW	0.8484	0.8596	0.8361	0.8540
Google News 300	0.8457	0.8437	0.8243	0.8447

**Table 3:** Topology of the LSTM model.

Layers	Parameters
Input	-
Embeding	-
LSTM	100, tahn
Dense	30, ReLU
Dropout	0.5
Output	1, sigmoid

**Table 4:** Effectivity of LSTM model with the topology presented in the Table 3.

LSTM	Precision	Recall	Accuracy	F1
64 SG	0.8764	0.8671	0.8628	0.8718
64 CBOW	0.8706	0.8648	0.8529	0.8677
30 SG	0.8722	0.8655	0.8543	0.8688
30 SBOW	0.8823	0.8364	0.8465	0.8587
120 SG	0.8636	0.8825	0.8568	0.8730
120 CBOW	0.8712	0.8655	0.8537	0.8684
200 SG	0.8943	0.8444	0.8576	0.8686
200 CBOW	0.8785	0.8697	0.8603	0.8741
Google News 300	0.8644	0.8305	0.8328	0.8471

**Table 5:** Topology of the CNN+LSTM model.

Layers	Parameters
Input	-
Embeding	-
Conv1D	30, 5, stride = 3, ReLU, padding = same
MaxPooling1D	pool_size = 2
Dropout	0.2
LSTM	100, tahn
Dense	30, ReLU
Dropout	0.5
Output	1, sigmoid

**Table 6:** Effectivity of CNN+LSTM model with the topology presented in the Table 5.

CNN+LSTM	Precision	Recall	Accuracy	F1
64 SG	0.8578	0.8617	0.8433	0.8598
64 CBOW	0.8426	0.8534	0.8293	0.8479
30 SG	0.8520	0.8620	0.8396	0.8570
30 SBOW	0.8399	0.8562	0.8288	0.8479
120 SG	0.8595	0.8589	0.8431	0.8592
120 CBOW	0.8531	0.8437	0.8319	0.8484
200 SG	0.8675	0.8669	0.8520	0.8672
200 CBOW	0.8380	0.8659	0.8319	0.8517
Google News 300	0.8438	0.8222	0.8160	0.8329

**Table 7:** Effectivity of the best models.

Best Models	Precision	Recall	Accuracy	F1
CNN 200 SG	0.8714	0.8644	0.8533	0.8679
CNN 200 SBOW	0.8484	0.8596	0.8361	0.8540
LSTM 200 SG	0.8943	0.8444	0.8576	0.8686
LSTM 200 CBOW	0.8785	0.8697	0.8603	0.8741
CNN+LSTM 200 SG	0.8675	0.8669	0.8520	0.8672
CNN+LSTM 200 CBOW	0.8380	0.8659	0.8319	0.8517

**Table 8:** Comparison of results our best model to the best models from previous studies focused on the same respectively similar problem.

Studies	Methods	Type of Detection	Best Results
[16]	BERT	Fake news (COVID/19)	F1 = 0.85
[17]	CNN, LSTM	Fake news	F1 = 0.97
[18]	CNN, LSTM	Fake accounts	Acc** = 0.93
[19]	BiLSTM	Cyberbullying	Acc** = 0.95
Our	LSTM, CBOW	Disinformations	Acc = 0.86, F1 = 0.87

**Table 9:** Significance analysis.

Topology	Topology	Statistics	*p*-Value	Decision
CNN	LSTM	−3.925	0.004	Different distributions (reject H0)
CNN	CNN+LSTM	1.545	0.161	Same distributions (fail to reject H0)
LSTM	CNN+LSTM	6.671	0.000	Different distributions (reject H0)

**Table 10:** List of standardized residuals.

W2V Representation	CNN	LSTM	CNN+LSTM
30 SG	1.0519	−0.1389	0.1167
30 CBOW	−1.4726	−1.5281	−0.9336
64 SG	0.9007	0.3965	0.5552
64 SBOW	−1.1008	−0.1322	−0.7772
120 SG	0.6784	0.7679	0.7526
120 CBOW	−0.4845	0.2560	−0.4301
200 SG	1.4242	0.6718	2.0317
200 CBOW	0.0000	1.3435	0.2257
Google News 300	−1.1062	−2.3740	−1.9945

## Data Availability

Dataset containing 27049 texts focused on disinformation about COVID-19 titled “COVID_dataset.csv” is available at (https://kristina.machova.website.tuke.sk/useful/) (accessed on 26 November 2022).

## References

[1] Gregor M., Vejvodová P. (2018). The Best Book about Fake News.

[2] Graham K.G. (2016). The Good German: Consensus and Dissent in the Development of British Wartime-Submisive Propaganda.

[3] Nocun N., Lamberty P. (2022). Fake Trust.

[4] Meta’s (formerly Facebook Inc.) Advertising Revenue Worldwide from 2009 to 2021. https://www.statista.com/statistics/271258/facebooks-advertising-revenue-worldwide/#professional.

[5] Wikforss A. (2021). Alternative Facts.

[6] Dawson A., Innes M. (2019). How Russia’s Internet Research Agency Built Its Disinformation Campaign.

[7] Schaeffer K. (2020). A Look at the Americans Who Believe There Is Some Truth to the Conspiracy Theory That COVID-19 Was Planned. https://www.pewresearch.org/fact-tank/2020/07/24/a-look-at-the-americans-who-believe-there-is-some-truth-to-the-conspiracy-theory-that-covid-19-was-planned/.

[8] Roman Y.M., Burela P.A., Pasupuleti V., Piscoya A., Vidal J.E., Hernandez A.V. (2022). Ivermectin for the Treatment of Coronavirus Disease 2019: A Systematic Review and Meta-analysis of Randomized Controlled Trials. Clin. Infect. Dis..

[9] Machova K., Mach M., Vasilko M. (2022). Comparison of Machine Learning and Sentiment Analysis in Detection of Suspicious Online Reviewers on Different Type of Data. Sensors.

[10] Vrysis L., Vryzas N., Kotsakis R., Saridou T., Matsiola M., Veglis A., Arcila-Calderón C., Dimoulas C. (2021). A Web Interface for Analyzing Hate Speech. Future Internet.

[11] Kandasamy V., Trojovský P., Al Machot F., Kyamakya K., Bacanin N., Askar S., Abouhawwash M. (2021). Sentimental Analysis of COVID-19 Related Messages in Social Networks by Involving an N-Gram Stacked Autoencoder Integrated in an Ensemble Learning Scheme. Sensors.

[12] Hrúz M., Gruber I., Knis J., Boháček M., Hlaváč M., Krňoul Z. (2022). One Model is not Enough: Ensembles for Isolated Sign Language Recognition. Sensors.

[13] Machova K., Mach M., Adamišin K. (2022). Machine Learning and Lexicon Approach to Texts Processing in Detection of Degrees of Toxicity in Online Discussions. Sensors.

[14] Atitalah S.B., Driss M., Almomani I. (2022). A Novel Detection and Multi-Classification Approach for IoT-Malware Using Random Forest Voting of Fine-Tuning Convolutional Neural Networks. Sensors.

[15] Islam M.R., Liu S., Wang X., Xu G. (2020). Deep Learning for Misinformation Detection on Online Social Networks: A Survey and New Perspectives. Soc. Netw. Anal. Min..

[16] Heidari M., Zad S., Hajibabaee P., Malekzadeh M., HekmatiAthar S., Uzuner O., Jones J.H. BERT Model for Fake News Detection Based on Social Bot Activities in the COVID-19 Pandemic. Proceedings of the IEEE 12th Annual Ubiquitous Computing, Electronics & Mobile Communication Conference (UEMCON).

[17] Umer M., Imtiaz Z., Ullah S., Mehmood A., Choi G.S., On B.W. (2020). Fake News Stance Detection Using Deep Learning Architecture (CNN-LSTM). IEEE Access.

[18] Sansonetti G., Gasparetti F., D’aniello G., Micarelli A. (2020). Unreliable Users Detection in Social Media: Deep Learning Techniques for Automatic Detection. IEEE Access.

[19] Raj C., Agarwal A., Bharathy G., Narayan B., Prasad M. (2021). Cyberbullying Detection: Hybrid Models Based on Machine Learning and Natural Language Processing Techniques. Electronics.

[20] Indurkhya N., Damerau F.J. (2010). Handbook of Natural Language Processing.

[21] Mikolov T., Chen K., Corrado G., Dean J. (2013). Efficient Estimation of Word Representations in Vector Space.

[22] Goodfellow I., Bengio Y., Courville A. (2016). Deep Learning.

[23] Manning C. Natural Language Processing with Deep Learning. http://web.stanford.edu/class/cs224n/slides/cs224n-2022-lecture16-CNN-TreeRNN.pdf.

[24] Hochreiter S., Schmidhuber J. (1997). Long Short-term memory. Neural Comput..

[25] Understanding LSTM Networks. http://colah.github.io/posts/2015-08-Understanding-LSTMs/.

[26] Glorot X., Bengio Y. Understanding the difficulty of training deep feedforward neural networks. Proceedings of the 13th International Conference on Artificial Intelligence and Statistics (AISTATS).

[27] Saxe A., McClelland J.L., Ganguli S. (2014). Exact Solutions to the Nonlinear Dynamics of Learning in Deep Linear Neural Networks. arXiv.

[28] El-kenawy E.M., Abutarboush H.F., Mohamed A.W., Ibrahim A. (2021). Advance Artificial Intelligence Technique for Designing Double T-shaped Monopole Antenna. Comput. Mater. Contin..

[29] Haase D., Amthor M. Rethinking depthwise separable comvolutions: How intra-kernel correlations lead to improved MobileNets. Proceedings of the IEEE Conference on Computer Vision and Pattern Recognition.

